# Seasonal evaluation of spermatogenesis of the hematophagous bat *Desmodus rotundus* in the Caatinga biome

**DOI:** 10.1371/journal.pone.0242932

**Published:** 2020-12-03

**Authors:** Soraia F. M. Silva, Laryssa C. A. Oliveira, Fernanda C. R. Dias, Eugenia Cordero-Schmidt, Juan C. Vargas-Mena, Ingrid G. M. Silva, Sônia N. Báo, João L. S. Luna, Ruthnaldo R. M. Lima, Raimundo F. A. Júnior, Naisandra B. S. Farias, Carlos E. B. Moura, Sérgio L. P. Matta, Danielle B. Morais

**Affiliations:** 1 Department of Morphology, Federal University of Rio Grande do Norte, Natal, Rio Grande do Norte, Brazil; 2 Department of General Biology, Federal University of Viçosa, Viçosa, Minas Gerais, Brazil; 3 Department of Ecology, Federal University of Rio Grande do Norte, Natal, Rio Grande do Norte, Brazil; 4 Department of Cell Biology, University of Brasília, Brasília, Distrito Federal, Brazil; 5 Department of Animal Sciences, Federal Rural University of the Semi-Arid Region, Mossoró, Rio Grande do Norte, Brazil; Universite Clermont Auvergne, FRANCE

## Abstract

This study was aimed to characterize the spermatogenic process and its seasonal variation in *Desmodus rotundus*, in the Caatinga biome, a water-limited ecosystem, with marked water restriction during most of the year. Collections of adult animals were performed during the dry and rainy seasons, and after euthanasia, their testes were processed histologically to perform morphological, morphometric, ultrastructural and immunohistochemical analyzes. The percentage of seminiferous epithelium, number of Leydig cells per gram of testis, and population of Sertoli cells and A-type spermatogonia presented by *D*. *rotundus* were significantly higher in the rainy season, while the percentage of lumen, mitotic index, support capacity performed by Sertoli cells, and overall yield of spermatogenesis were higher in the dry season. The ultrastructure of spermatogenesis was similar to that described in other mammals, and the immunohistochemical analysis revealed activity of the aromatase enzyme in Sertoli cells, Leydig cells, spermatocytes and spermatids, as well as the presence of androgen receptors in Sertoli cells and Leydig cells. FGF2 activity was detected in primary spermatocytes in zygotene and pachytene, as well as secondary spermatocytes and rounded and elongated spermatids, while the BCL-2 protein was expressed in primary spermatocytes in zygotene and pachytene, secondary spermatocytes, and rounded spermatids. The activity of these molecules was similar in both seasons, and associated with the morphometric findings, indicates maintenance in the integrity of the seminiferous epithelium throughout the year. The seasonal study of *D*. *rotundus* spermatogenesis indicates a continuous spermatogenesis pattern and suggests a greater production of spermatozoa in the rainy season in the Caatinga biome.

## Introduction

*Desmodus rotundus* is the most abundant species among vampire bats, appearing exclusively in Latin America, from Mexico to Argentina, Chile and Uruguay, and the entire Brazilian territory [1]. This species has an exclusively hematophagous feeding habit, with a preference for the blood of large domestic or wild mammals, making it a potential vector of the rabies virus [2–4]. Their close proximity to cattle, where they find an abundance of food resources, and promiscuous mating system, are some of the factors allowing this species to reproduce throughout the year [2, 5]. There is no significant influence of temperature or availability of food on their reproductive abilities, when compared to other neotropical bats [6, 7].

Despite its presence over a wide area and the numerous studies on its ecology and physiology adapted to hematophagy, only few studies have addressed the reproductive aspects of the male *D*. *rotundus*, particularly in Caatinga, a seasonally dry forest with a semi-arid climate, located in Northeastern Brazil. This is a highly anthropized biome, where the greatest availability of forage for cattle occurs in the rainy season. The dry season in this region is severe and usually prolonged. Upon the onset of drought, senescent leaves of woody plants are incorporated into the diet of these animals and may represent the only forage resource available to cattle in some areas. Thus, the difficulty in managing livestock during the dry season can cause a decrease in herds, consequently affecting the diet of hematophagous bats [8, 9]. It is not known, however, whether such limitations affect the reproductive performance of *D*. *rotundus*.

Although testicular histology of this vampire bat species is well studied, the seasonal behavior of the cells that make up the testicular parenchyma and the rates of sperm production in this species have not been evaluated, especially in a region that experiences a severe period of water restriction [9]. Considering the influence of climatic factors on bat reproduction [7, 10], it is important to obtain information regarding the same, as that would support the development of population control measures, bearing in mind the ecological importance of the species and their economic and epidemiological impact, both on livestock and on human health.

It is also important to emphasize the lack of information about the reproductive abilities of mammals in general, in the Caatinga biome [11]. Thus, the objective of this study was to characterize the spermatogenic process of *D*. *rotundus* captured in this region, considering the morphological, morphometric, immunohistochemical and ultrastructural aspects of the testes, from the study of specimens captured at different periods of the year.

## Material and methods

### Study area and animal collection

The animals were collected in Lajes city, Rio Grande do Norte, Brazil (05°42'00"S, 36°14'41"W). These captures were authorized by the Chico Mendes Institute for Biodiversity Conservation (ICMBio, license number 55562–1). All experimental procedures were conducted in accordance with the recommendations of the National Council for Animal Experimentation Control (CONCEA). The protocol was approved by the Ethics Committee on Animal Use of the Federal University of Rio Grande do Norte (CEUA UFRN, protocol number 056/2016). All efforts were made to minimize animal suffering.

Two annual seasons were established for collections: one dry, from September to February, and one rainy, from March to August [12]. Thus, the specimens were captured seasonally and the dry season (n = 7) and rainy season (n = 7) groups were established. For each season, the captures occurred on the same day, respectively on the months of January and July for the dry and rainy seasons. Specimens of male and adult *D*. *rotundus* were captured at nightfall using mist nets at the entrance to the abandoned ore galleries, which animals used as shelters. Adult animals were identified based on the observation of fusion of the epiphyseal cartilage of the fourth finger at the metacarpal-phalangeal junction [13]. At dry and rainy seasons, respectively, eight and seven adult females were also captured, and in each season, five of them were pregnant.

The animals were anesthetized intraperitoneally (xylazine 50 mg/kg and ketamine 80 mg/kg), followed by weighing of the animals and removal of the reproductive system. Subsequently they were euthanized by deepening the anesthetic plane (xylazine 150 mg/kg and ketamine 240 mg/kg).

### Histological processing

One testis of each animal was fixed in Karnovsky solution [14] for 24 hours and histologically processed for either morphological and morphometric analyses under light microscopy, or ultrastructural analysis, under transmission electron microscopy. Testicular fragments were embedded in glycol methacrylate (Historesin, Leica), sliced into 3 μm sections using a rotatory microtome (Leica RM 2245), and stained with toluidine blue/sodium borate 1% (Merck) for light microscopy analyses.

For ultrastructural analysis, testicular fragments were post-fixed with 2% osmium tetroxide and 1.6% potassium ferricyanide in 0.2 M sodium cacodylate buffer, followed by overnight staining in 0.5% aqueous solution of uranyl acetate. Dehydration was performed in ethanol and acetone, followed by the addition of embedding resin (Spur, Sigma-Aldrich^®^). Ultrathin sections were contrasted with uranyl acetate and lead citrate and observed under a transmission electron microscope (JEOL 1011).

The other testis of each animal was fixed in 4% paraformaldehyde and processed for inclusion in histological paraffin for subsequent immunohistochemical analyses, aiming the knowledge of the cellular activity at a molecular level. So, we investigated seasonally the activity of the pre-apoptotic protein BCL-2 and of the fibroblast growth factor (FGF2), to check the presence or absence of testicular regression on the seasons evaluated, as well the enzyme aromatase and androgen receptors, to access the hormonal capacity along the seasons. So, testicular sections with 4-μm thickness were obtained on signaled slides. The histological sections were deparaffinized, rehydrated, washed in 0.3% Triton X-100 in phosphate buffer and incubated with endogenous peroxidase (3% hydrogen peroxide). The sections were incubated overnight at 4°C in the presence of primary antibodies (Santa Cruz Biotechnology, Inc. EUA) against pre-apoptotic protein BCL-2 (1: 400), fibroblast growth factor (FGF2, 1: 400), aromatase (1: 200), and androgen receptor (1: 200). The sections were carefully rinsed with phosphate buffer and incubated in the presence of secondary antibody streptavidin/HRP-conjugated (Biocare Medical, USA) for 30 minutes. Immunoreactive cells were visualized by colorimetric detection following the manufacturer’s protocol (TrekAvidin-HRP Label + Kit Biocare Medical, Dako, USA). The sections were counterstained with hematoxylin and positive marked areas were captured by a photomicroscope (Nikon E200 LED).

The number of positive cells per tubular cross section for each antibody was quantified in relation to the number of cells without immunostaining, in an area of approximately 40,000 μm^2^. The following formula was used: [(number of marked cells / number of unmarked cells) / number of analyzed sections] [15].

### Testicular morphometry

Both testes were weighed after fixation, using an analytical balance (BEL M214AIH). The gonadosomatic index (GSI) was calculated by dividing the testes weight by body weight and multiplying by 100, to quantify the weight proportion of the testicles with respect to the total body mass [15].

Digital images were obtained using a light-field photomicroscope (Olympus BX-50 or BEL Bio2/3 Eurekam 5.0) and analyzed based on testicular stereology, using the Image-Pro Plus^®^ software. The volumetric proportions of all components of the seminiferous tubule (tunica propria, seminiferous epithelium, and lumen), intertubule and tunica albuginea were determined after counting 3,520 intersection points, per animal, in 10 square grids randomly placed over the digital images (100x magnification) [15, 16].

The seminiferous tubules volume (STV) is a function of the volume of the testis and the volumetric proportion of these tubules in the testicular parenchyma, and was used to calculate the tubulosomatic index (TSI), which quantifies the mass of seminiferous tubules as a proportion of the total body mass. Thus, the TSI was obtained by dividing the STV by body weight and multiplying the result by 100. The mean tubular diameter was obtained by measuring 20 tubular cross-sections per animal, which presented the most circular shape, regardless of the stage of the cycle. These sections were also used to measure the height of the seminiferous epithelium, from the tunica propria to the tubular lumen, taking two diametrically opposite measurements in each cross section. The seminiferous tubule length (STL, in meters) per testis was estimated as follows: STL = STV/ πR^2^ (πR^2^ = tubule area; R = tubular diameter/2). The STL was divided by the testicular weight to calculate the length of the seminiferous tubules per gram of testis (STL/g), to allow comparisons between different species [15–17].

Coincident points (n = 1000) over the intertubular components were recorded: Leydig cell, blood and lymphatic vessels, and connective tissue. The volumetric rates of these components were also estimated (400x magnification). The percentage of these components in the intertubule was estimated by multiplying the total number of points on each component by 100 and dividing the obtained value by 1000. The percentage of these components in the testis was obtained by multiplying the percentage of intertubular components by the percentage of each component in the intertubule and dividing the obtained value by 100. The volume of each intertubular component in the testicular parenchyma was calculated by the following formula: (percentage of each component in the testis x gonadal weight) / 100. The values were expressed in μL [15, 16, 18]. Since the mammalian testis density is around 1 [19], its weight was considered equal to the volume.

Leydig cells morphometry was performed, since they are the cells responsible for the production of testosterone, and therefore play a fundamental role in testicular activity. The mean diameter of the Leydig cell was obtained after measuring 30 cells per animal, and selecting those with the most spherical nuclei and evident nucleoli. The nuclear volume was obtained by using the formula 4/3πR^3^ (R = nuclear diameter/2). The cytoplasmic volume was estimated by multiplying the percentage of cytoplasm by the nuclear volume, divided by the nuclear percentage. The single cell volume was estimated by adding the nuclear volume to the cytoplasmic volume. These values were expressed in μm^3^. The total volume occupied by the Leydig cells in the testicular parenchyma was obtained by multiplying the percentage of Leydig cells in the testis by the gonadal weight and dividing the obtained value by 100. The number of Leydig cells per testis was estimated from the Leydig cell individual volumes and the total volume occupied by these cells in the testicular parenchyma. This value was divided by the total gonadal weight to estimate the number of Leydig cells per gram of testis. The Leydigosomatic index (LSI), which quantifies the mass of Leydig cells as a proportion of the total body mass, was estimated by dividing the Leydig cell volume in the testicular parenchyma by the body weight and multiplying by 100 [15, 16, 18].

### Quantification of spermatogenic yield

Since the analysis of the cells that composes the stage 1 of the seminiferous epithelium cycle (SEC) allows the quantification of spermatogenic indexes, the number of each one of these cells was estimated by counting their nuclei (germ cells) or nucleoli (Sertoli cells) in 10 random tubular cross sections per animal. Thirty nuclear diameters of A-type spermatogonia (A), primary spermatocytes in preleptotene/leptotene (PL/L), primary spermatocytes in pachytene (P), rounded spermatids (RS) and Sertoli cells (SC) nuclei were measured for each animal. The results were corrected due to variations in the size of the cells and the section thickness, as described by Amann and Almquist [20].

The intrinsic yield of spermatogenesis was calculated based on the ratio between corrected germ cell numbers, in order to quantify spermatogenesis efficiency. The mitotic index (PL-L: A) was calculated to determine the loss or degeneration that occurred during the spermatogonial phase; the meiotic index (RS: P), to determine the efficiency of the meiotic divisions; and the overall yield of spermatogenesis (RS: A) to quantify the efficiency of the spermatogenic process [16, 17].

The total support capacity of Sertoli cell was also determined, indicating the ability of these cells to support the total number of germ cells [(A + PL-L + P + RS): SC]. The total number of Sertoli cells per testis was obtained by multiplying their corrected number by the tubular length per testis (in μm) and dividing the result by the section thickness [19]. The obtained results were divided by the testicular weight in order to calculate the number of Sertoli cells per gram of testis [16, 17].

The cell loss in spermiogenesis was assumed to be insignificant [21] and the spermatic reserve of the testis (SRT) was calculated based on the round spermatid populations, using the formula: [(seminiferous tubule length / cut thickness) × corrected number of round spermatids per cross-section] [22].

The daily spermatic production (DSP) was estimated based on the knowledge of the duration of a CES of *D*. *rotundus*, estimated at 8.23 days [16]. Thus, DSP was calculated by dividing the SRT obtained in the present study by 8.23, according to the formula proposed by Amann [23]. Both the SRT and the DSP per gram of testis were obtained by dividing its values per testis by the testes’ weight [17].

### Statistical analysis

The morphometric parameters analyzed were compared between seasons, and the variables were submitted to Student’s t-test, considering a significance level of 5% (p≤ 0.05), using the SPSS software version 12.0 for Windows (SPSS Inc.; Chicago, IL, USA). The results were expressed as mean ± standard deviation. The immunohistochemical analysis results were submitted to descriptive statistical analysis and expressed as mean ± standard deviation.

## Results

### Biometry and morphometry of seminiferous tubules

**Fig 1:**
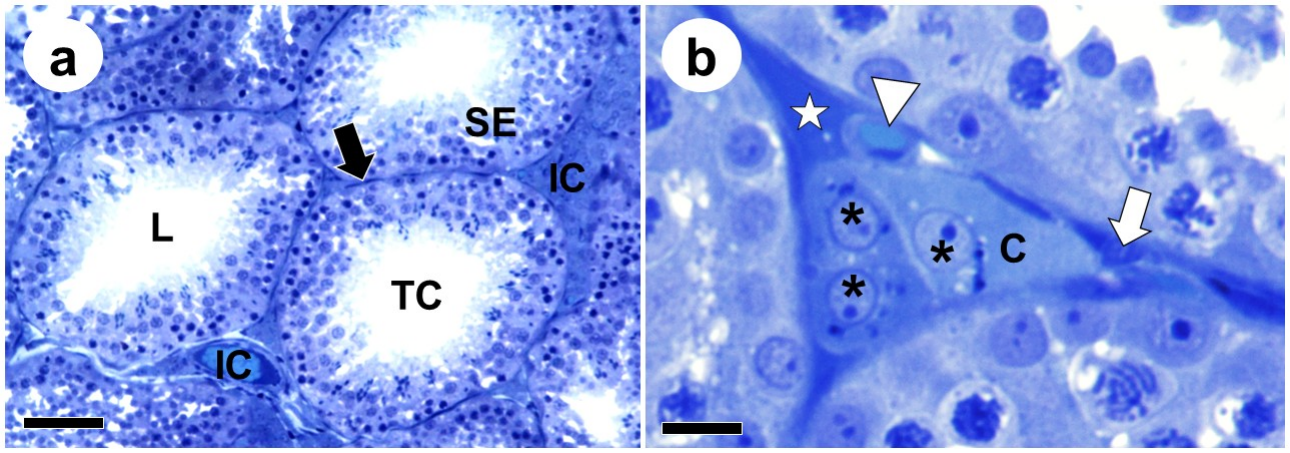
Cross sections of *Desmodus rotundus* testicular parenchyma. **TC:** Tubular compartment; **SE:** Seminiferous epithelium; **L:** Lumen of seminiferous tubule; **➡:** Tunica propria; **IC:** Intertubular compartment; *: Leydig cell nuclei; **C:** Leydig cell cytoplasm; ☆: Lymphatic vessel; **∇:** Blood vessel; **⇨:** Connective tissue. Bars: a: 70 μm; b: 30 μm.

The arrangement of the testicular parenchyma of *D*. *rotundus* is shown in Fig 1. Table 1 shows the average values referring to biometrics and volumetric proportions of the components of testicular parenchyma, in the dry and rainy seasons. Considering the average of the two seasons, the animals had an average body weight of 33.51 g and an average testicular weight of 0.19 g, resulting in an average GSI of 0.56%. The testicular parenchyma was composed predominantly of seminiferous tubules, of which 4.37% were represented by tunica propria, 69.31% by the seminiferous epithelium and 22.01% by the lumen. There was a statistically significant difference between seasons in relation to the percentages of the tubular compartment represented by seminiferous epithelium and lumen, so that the percentage of seminiferous epithelium was significantly higher in the rainy season. Consequently, the lumen percentage was significantly higher in the dry season.

**Table 1:** Biometry, morphometry and stereology of the testicular components of *Desmodus rotundus*, during dry and rainy seasons.

Parameters	Dry season	Rainy season	Anual mean
Body weight (g)	33.02±5.22	34.01±1.52	33.51±3.37
Testes weight (g)	0.18±0.04	0.20±0.04	0.19±0.04
Gonadosomatic index (%)	0.53±0.10	0.58±0.10	0.56±0.10
Tunica albuginea (%)	10.49±2.40	10.13±1.68	10.31±2.04
Seminiferous tubules percentage (%)	95.76±1.01	95.63±1.60	95.69±1.31
Seminiferous tubules volume (mL)	0.17±0.04	0.19±0.04	0.18±0.04
Tunica propria (%)	4.23±0.93	4.50±0.68	4.37±0.80
Seminiferous epithelium (%)	66.59±6.10^b^	72.04±2.39^a^	69.31±4.25
Lumen (%)	24.94±4.99^a^	19.09±1.81^b^	22.01±3.40
Intertubule percentage (%)	4.24±1.01	4.38±1.60	4.31±1.31
Intertubulae volume (mL)	0.007±0.002	0.009±0.003	0.008±0.002
Tubulesomatic index (%)	0.51±0.10	0.56±0.10	0.53±0.10
Tubular diameter (μm)	441.37±26.70	422.97±38.46	432.17±32.58
Seminiferous epithelium height (μm)	97.58±15.29	84.94±8.62	91.26±11.95
Seminiferous tubules length per testis (m)	1.10±0.27	1.37±0.30	1.23±0.29
Seminiferous tubules length per gram of testis (m/g)	6.32±0.71	6.98±1.41	6,65±1.06

Lines with different superscripts indicate statistical difference between values, according to t-test (p<0.05). Data are expressed by mean ± standard deviation.

There was no significant variation between seasons for the other parameters. Thus, the average tubular volume between the two seasons was 0.18 ml, resulting in an average TSI of 0.53%. The tubular diameter and height of the seminiferous epithelium presented mean values of 432.17 μm and 91.26 μm, respectively. The animals in this study had 1.23 m of seminiferous tubules per testicle and 6.65 m of seminiferous tubules per gram of testis.

### Quantification of spermatogenic yield

Table 2 shows the average per season of the corrected number of the cell types found in stage 1 of the SEC, as well as the indices of sperm production in *D*. *rotundus*. The count of spermatogonia and Sertoli cells was significantly higher during the rainy season, as well as the number of Sertoli cells per testis and per gram of testis. On the other hand, the mitotic index, the general spermatogenic yield and the support capacity performed by Sertoli cells were higher during the dry season.

**Table 2:** Germ cell population in stage 1 of the seminiferous epithelium cycle and sperm production indexes of *Desmodus rotundus*, during dry and rainy seasons.

Parameters	Dry season	Rainy season	Anual mean
Sertoli cell	2.29±0.52^b^	3.51±0.74^a^	2.90±0.63
A-type spermatogonia	0.67±0.24^b^	1.00±0.12^a^	0.84±0.18
Primary spermatocyte in Pre-Leptotene/Leptotene	16.58±3.11	16.32±3.54	16.45±3.32
Primary spermatocyte in Pachytene	18.19±3.05	16.89±2.23	17.54±2.64
Rounded spermatid	58.90±14.47	49.24±7.28	54.07±10.87
Mitotic index	25.43±4.66^a^	16.50±4.59^b^	20.97±4.63
Meiotic index	3.23±0.42	2.93±0.37	3.08±0.39
Spermatogenic yield	90.79±19.32^a^	49.87±10.90^b^	70.33±15.11
Sertoli cell support capacity	44.71±22.55^a^	24.57±5.24^b^	34.64±13.90
Sertoli cell number /testis (x10^6^)	8.34±2.43^b^	15.31±3.49^a^	11.83±2.96
Sertoli cell number /g of testes (x10^6^)	4.86±1.26^b^	8.07±3.28^a^	6.46±2.27
Spermatic reserve (x10^6^)	21.55±7.06	23.23±6.17	22.39±6.62
Spermatic reserve/g of testis (x10^7^)	12.22±1.56	11.64±1.64	11.93±1.60
Daily spermatic production (x10^5^)	26.18±8.58	28.22±7.50	27.20±8.04
Daily spermatic production/g of testis (x10^6^)	14.85±1.89	14.14±2.00	14.49±1.94

Lines with different superscripts indicate statistical difference between values, according to t-test (p<0.05). Data are expressed by mean ± standard deviation.

The animals had an average spermatic reserve per gram of testis of 11.93 x 10^7^ cells, and an average daily spermatic production per gram of testicle of 14.49 x 10^6^ cells was estimated. These values remained unchanged significantly throughout the year.

**Fig 2:**
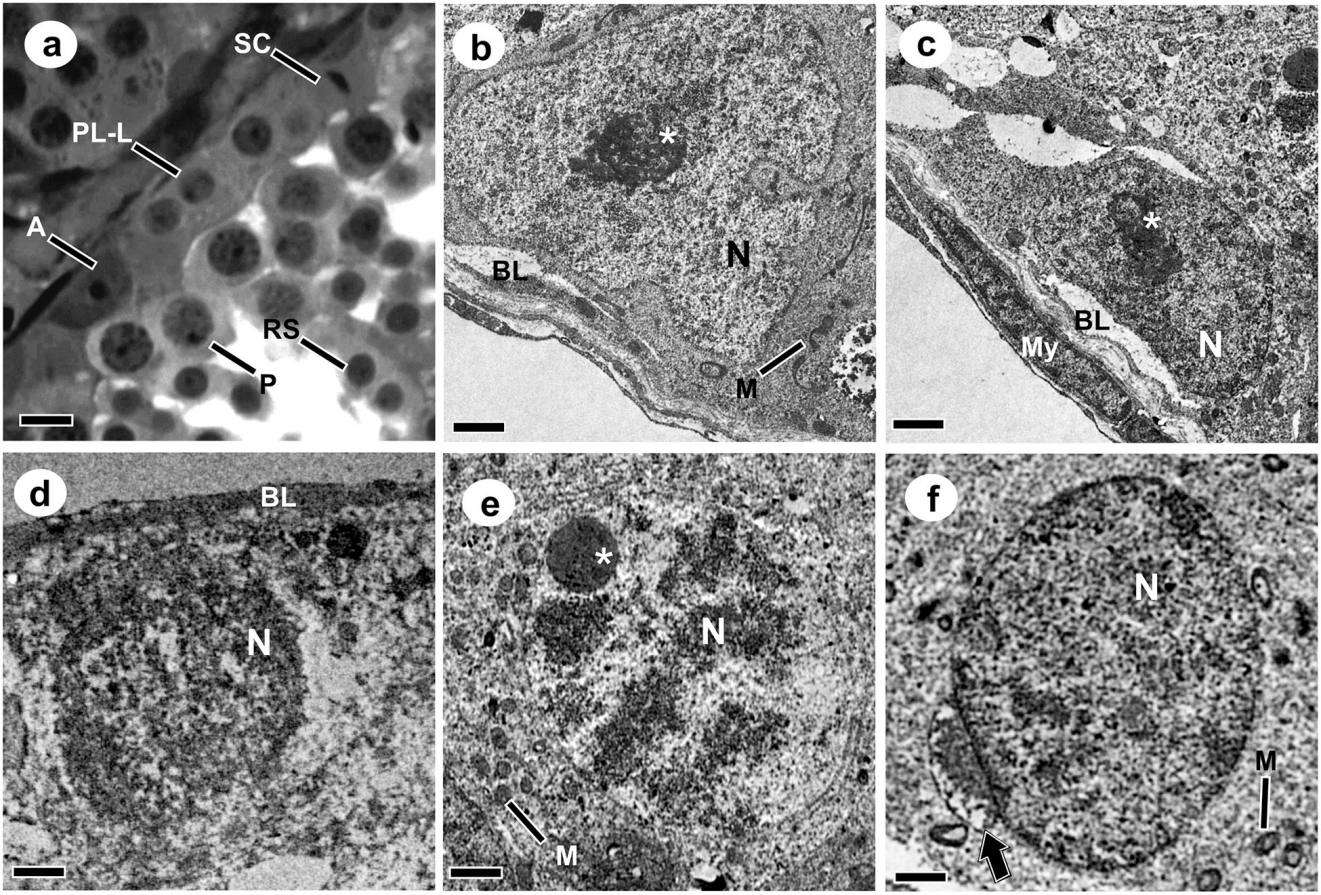
Cross sections of *Desmodus rotundus* testes showing the cells that composes the stage 1 of the seminiferous epithelium cycle, under light (a) and transmission electron microscopy (b-f). **a:** A-type spermatogonia (**A**); Primary spermatocyte in Pre-Leptotene/Leptotene (**PL-L**); Primary spermatocyte in Pachytene (**P**); Rounded spermatid (**Ar**); Sertoli cell (**SC**). **b:** Sertoli cell; **c:** A-type spermatogonia; **d:** Primary spermatocyte in Pre-Leptotene/Leptotene; **e:** Primary spermatocyte in Pachytene; **f:** Rounded spermatid. **N:** Nucleus; *: Nucleoli; **BL:** Basal lamina; **M:** Mitochondria; **My:** Myoid cell; **➡:** Acrossome. Bars: a: 10 μm; b-f: 2 μm.

The ultrastructure of the cell types that make up the germinative epithelium at stage 1 of the SEC, as well as their organization under light microscopy is shown in Fig 2a. Sertoli cells with characteristic nuclei and fragmented nucleoli were evident, located close to the basal lamina (Fig 2b). A-type spermatogonia were elliptical in shape, with an oval nucleus containing granular chromatin with a large, granular and irregular, centrally located single nucleolus. These cells were firmly attached to the basal lamina, with which they formed projections and depressions (Fig 2c).

Primary spermatocyte nuclei in preleptotene/leptotene showed regions of linear chromatin, located close to the basal lamina (Fig 2d). The primary spermatocytes in pachytene were located in the median region of the epithelium, and presented a compact and dense nucleolus (Fig 2e). Rounded spermatids were observed close to the lumen of the seminiferous tubules, with spherical and regular nuclei, containing filamentous clusters of chromatin. The nucleolus was visible in the initial spermatids. However, with the entry of these in the process of spermiogenesis, the nucleolus was disorganized, and no nucleolar material was observed in the next phases. Under the nuclear surface of these cells, it was still possible to visualize the beginning of the association with the acrosomal cap (Fig 2f).

**Fig 3:**
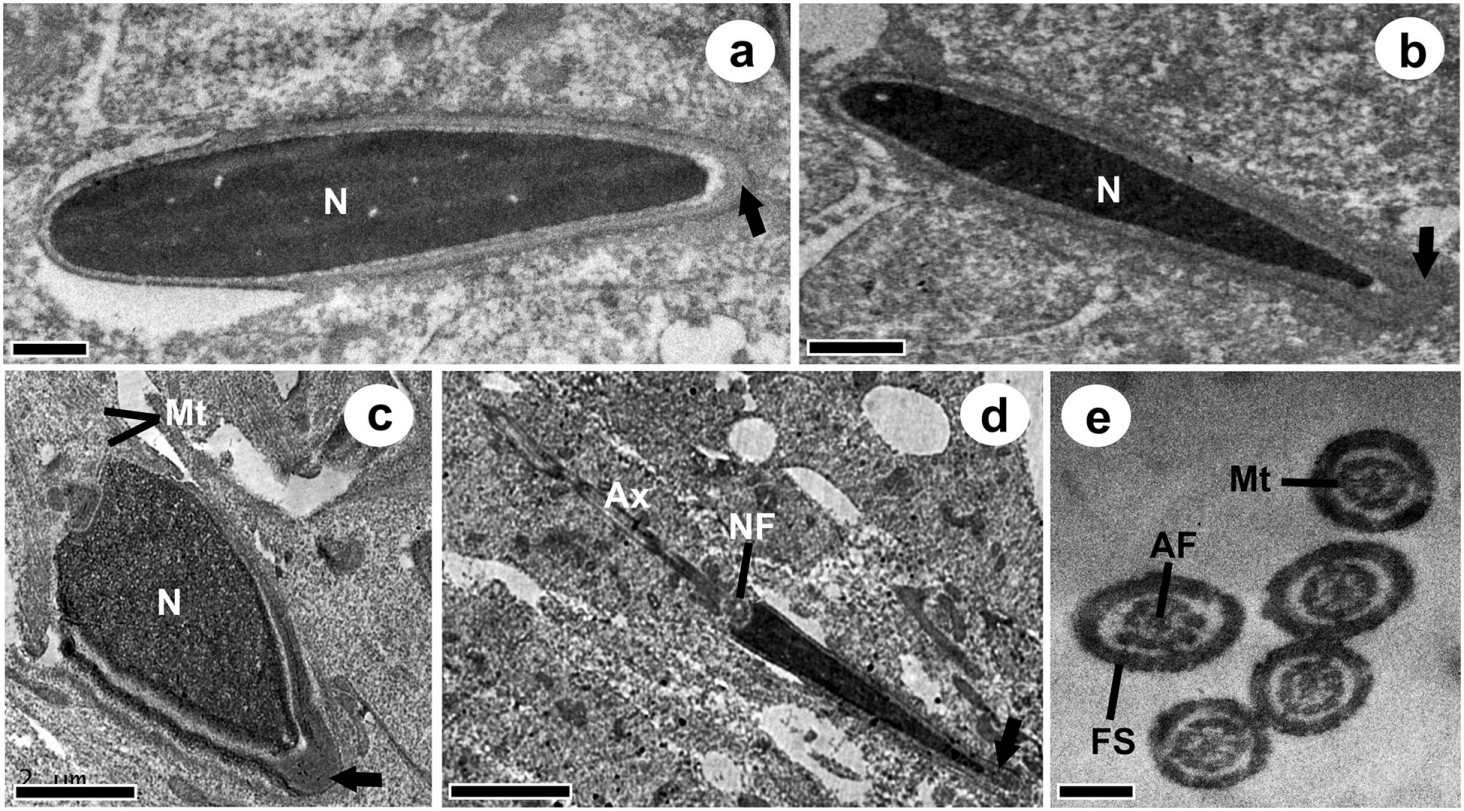
Ultrastructural aspects of spermiogenesis in *Desmodus rotundus*. **a-c:** elongated spermatids in the final stages of acrossomal cap formation; **d:** late spermatid beggining the organization of the intermediate part of the sperm tail; **e**: cross sections of flagella. **N:** Nucleus; **➡:** Acrossomal cap; **Mt:** Microtubules; **Ax:** Axoneme; **NF:** Nuclear fossa; **FS:** Fibrous sheath; **FA:** Axial filament. Bars: a: 0,5 μm; b: 1 μm; c-d: 2 μm; e: 0,5 μm.

Fig 3 shows the ultrastructure of the elongated spermatids and *D*. *rotundus*’ spermatozoa. The main changes observed during the spermiogenesis phase were the major elongation and compacting of the nucleus of future sperm, the development of the acrosome (Fig 3a–3c) and the flagellum (Fig 3d and 3e). The axoneme forms at the portion end of the spermatid nucleus (Fig 3d), while the acrosome condenses in its cranial portion. The presence of perforatorium in this region was not detected. The nuclear fossa, which allows the development of the flagellum, can be seen in Fig 3d, while the microtubules can be seen in Fig 3c and 3e. These were organized in an axial filament composed of a pair of central microtubules surrounded by 9 pairs of peripheral microtubules, composing the axoneme. These structures are surrounded by a fibrous sheath. All the ultrastructural aspects of the cells evaluated were similar between the two seasons.

### Morphometry and morphology of the intertubular compartment

Table 3 shows the average values for the morphometry of the intertubular compartment in the testes of *D*. *rotundus*. This compartment was composed of Leydig cells, blood vessels, lymphatic vessels and connective tissue (Fig 1b). Leydig cells were the predominant elements in the two seasons, representing on average 67.26% of the intertubule and 2.87% of the testicular parenchyma as a whole. None of the intertubular components showed significant variation between seasons.

**Table 3:** Volumetric proportion (%) and volume of the intertubular compartment of *Desmodus rotundus*, during dry and rainy seasons.

Parameters	Dry season	Rainy season	Anual mean
Percentage in the intertubule (%)			
Leydig cell	69.63±8.45	64.89±12.84	67.26±10.65
Blood vessel	17.06±8.40	23.54±11.20	20.30±9.80
Lymphatic vessel	7.79±4.42	6.77±3.93	7.28±4.17
Connective tissue	5.53±1.69	4.80±2.89	5.16±2.29
Percentage in the testicular parenchyma (%)			
Leydig cell	2.90±0.55	2.83±1.06	2.87±0.80
Blood vessel	0.74±0.42	1.01±0.59	0.88±0.51
Lymphatic vessel	0.35±0.24	0.32±0.27	0.33±0.25
Connective tissue	0.24±0.12	0.21±0.15	0.23±0.13
Volume in the testicular parenchyma (μL)			
Leydig cell	5.01±1.18	5.58±2.20	5.29±1.69
Blood vessel	1.25±0.68	1,95±1.08	1.60±0.88
Lymphatic vessel	0.60±0.38	0.59±0.45	0.59±0.42
Connective tissue	0.42±0.17	0.42±0.28	0.42±0.22

Lines with different superscripts indicate statistical difference between values, according to t-test (p<0.05). Data are expressed by mean ± standard deviation.

**Fig 4:**
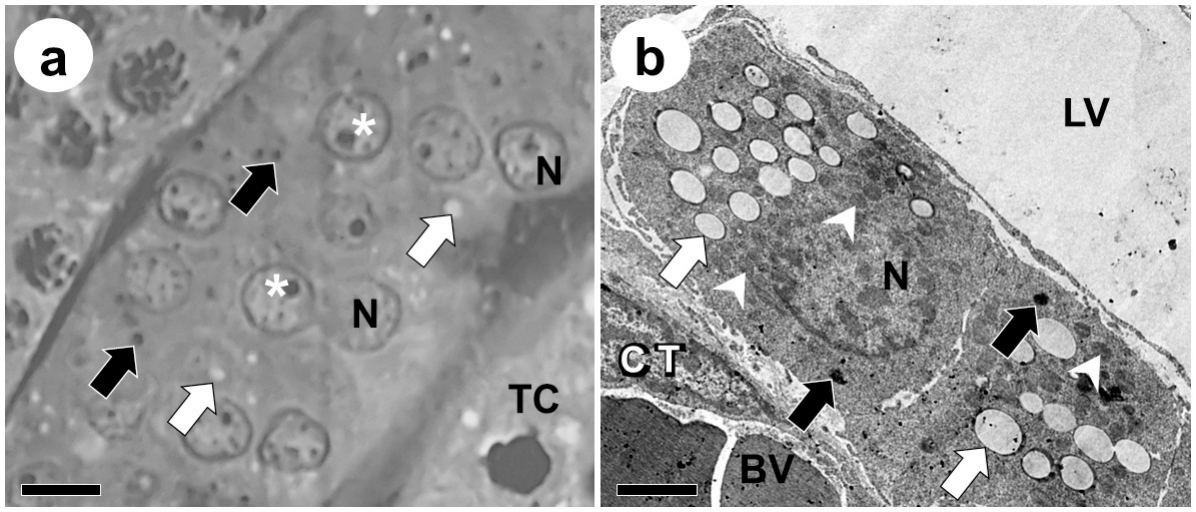
Leydig cell morphology in *Desmodus rotundus* testes under light (a) and transmission electron microscopy (b). **N:** Leydig cell nuclei; *: Leydig cell nucleoli; **⇨:** Lypidic droplets on Leydig cell cytoplasm; **➡:** Lipofuscin granules on Leydig cell cytoplasm; ➤: Mitochondria on Leydig cell cytoplasm; **LV:** Lymphatic vessel; **BV:** Blood Vessel; **CT:** Connective tissue; **TC:** Tubular compartment. Barras: a: 15 μm; b: 7,5 μm.

The averages for the morphometry of Leydig cells from *D*. *rotundus* are shown in Table 4, and their morphology is shown in Fig 4. These cells had an ellipsoid nucleus, lipid droplets and lipofucsin granules dispersed throughout the cytoplasm. Abundant mitochondria were also observed (Fig 4b). There were no significant differences in Leydig cells’ percentages and nuclear and cytoplasmic volumes between seasons, as well as in their individual and total volumes and LSI. On the contrary, the total number of Leydig cells per gram of testis was higher during the rainy season, where about 48.14 x 10^5^ cells were found.

**Table 4:** Seasonal morphometry of Leydig cell in the bat *Desmodus rotundus*.

Parameters	Dry season	Rainy season	Anual mean
Nuclear diameter (μm)	15.31±0.96	15.72±3.38	15.51±2.17
Nuclear percentage (%)	27.02±12.34	25.51±11.73	26.27±12.03
Nuclear volume (μm^3^)	1896.75±349.83	2301.70±1801.30	2099.22±1075.56
Cytoplasmic percentage (%)	72.98±12.34	74.49±11.73	73.73±12.03
Cytoplasmic volume (μm^3^)	6097.88±2543.90	7653.70±5171.65	6875.79±3857.77
Leydig cell volume (μm^3^)	7994.63±2795.80	9955.40±6813.34	8975.01±4804.57
Leydig cell number / testis (x10^5^)	7.99±4.69	9.67±6.08	8.83±5.38
Leydig cell number /g of testis (x10^5^)	47.28±29.92^b^	48.14±25.57^a^	47.71±27.74
Leydigosomatic index (%)	0.015±0.004	0.016±0.006	0.016±0.005

Lines with different superscripts indicate statistical difference between values, according to t-test (p<0.05). Data are expressed by mean ± standard deviation.

### Immunohistochemical analyses

**Fig 5:**
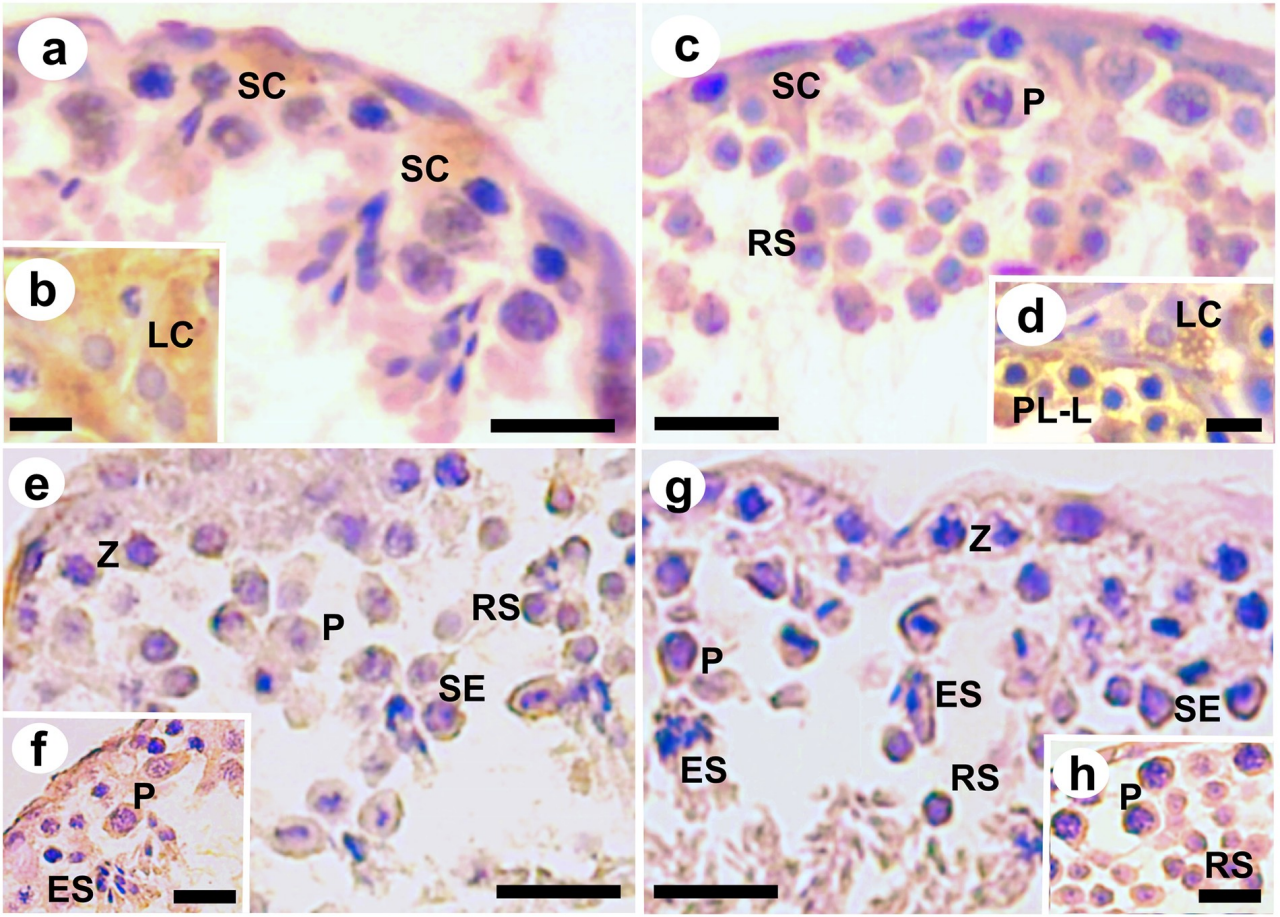
Imunohistochemical detection of Androgen receptors (a-b), Aromatasis (c-d), FGF2 (e-f) e BCL-2 (g-h) in the testes of *Desmodus rotundus*. **SC:** Sertoli cell; **LC:** Leydig cell; **PL-L:** Primary spermatocyte in transition from Pre-Leptotene to Leptotene; **Z:** Primary spermatocyte in Zygotene; **P:** Primary spermatocyte in Pachytene; **SE:** Secondary spermatocyte; **RS:** Rounded Spermatid; **ES:** Elongated Spermatid. Bars: a-d: 30 μm; e-h: 40 μm.

Androgen receptors (Fig 5a and 5b) were equally distributed in the nuclei of Sertoli cells between the dry and rainy seasons, while the distribution of these receptors in the cytoplasm of Leydig cells was observed only in the rainy season (Table 5). Aromatase (Fig 5c and 5d) had a homogeneous distribution in both stations, both in the nuclei of Sertoli cells and in the cytoplasm of Leydig cells, primary spermatocytes in pachytene and rounded spermatids. However, more frequent labeling for this enzyme was observed in the cytoplasm of primary spermatocytes in the preleptotene/leptotene transition in the rainy season (Table 5).

**Table 5:** Expression frequency of the androgen receptor, aromatase, FGF 2 and BCL-2 in the testes of *Desmodus rotundus*, during dry and rainy seasons.

Cell type	Season	Androgen	Aromatase	FGF 2	BCL-2
Sertoli cell	Dry	1.00±0.22	1.00±0.32	---	---
	Rainy	1.19±0.34	1.00±0.50	---	---
Leydig cell	Dry	---	1.00±0.46	---	---
	Rainy	0.88±0.40	1.00±1.17	---	---
Primary spermatocyte in Pre-Leptotene/Leptotene	Dry	---	1.00±0.55	---	---
	Rainy	---	6.67±2.64	---	---
Primary spermatocyte in Zygotene	Dry	---	---	1.00±1.22	1.00±0.25
	Rainy	---	---	1.00±0.47	1.00±0.66
Primary spermatocyte in Pachytene	Dry	---	1.00±0.48	1.00±0.49	1.00±0.31
	Rainy	---	1.00±1.00	1.00±0.16	1.00±0.23
Secondary spermatocyte	Dry	---	---	1.00±1.00	1.00±1.00
	Rainy	---	---	---	---
Rounded spermatid	Dry	---	1.00±0.53	1.00±0.36	1.00±0.18
	Rainy	---	1.11±1.16	1.00±0.51	1.00±0.00
Elongated spermatid	Dry	---	---	1.00±0.75	---
	Rainy	---	---	1.00±0.30	---

Data are expressed by mean ± standard deviation.

The activity of FGF2 (Fig 5e and 5f) and BCL-2 (Fig 5g and 5h) was found to be similar between the dry and rainy seasons in the cytoplasm of primary spermatocytes in zygotene and pachytene, secondary spermatocytes and rounded spermatids (Table 5). The elongated spermatids also showed a similar pattern of immunostaining for FGF2 in both seasons.

## Discussion

*D*. *rotundus* is a species restricted to the Americas, with an intriguing hematophagous eating habit, the only species specialized in the consumption of the blood of mammals, and the most abundant vampire bat species. Some morphological studies have been conducted to better understand its reproductive cycle, aiming at both its conservation and a rational management of the species. This study therefore represents an additional effort towards understanding the seasonal reproduction of this species in a region that faces scarcity of water and food resources during a considerable period of the year, focusing on the testicular morphology of males, since studies on spermatogenesis are scarce. The presence of pregnant females observed in the collection area during the whole year suggests a continuous reproductive cycle for the species, despite some of the spermatogenic parameters evaluated shows greater values in one season or another, as we will discuss below.

### Biometry and morphometry of seminiferous tubules

The GSI obtained was similar to that previously registered for the species in an area of Atlantic forest (0.54%; [16]), and higher than that found in other bat species, such as the frugivore *Sturnira lilium* (0.27%; [17]) and in the insectivore *Molossus molossus* (0.47%; [24]). Its GSI reflects a greater investment in testicular mass in individuals of the order Chiroptera when compared to other mammalian orders, which is consistent with the need for maintenance of the harem, since polygyny being the social organization commonly found among bats [25–27].

The highest percentage of seminiferous epithelium observed in the rainy season led directly to the lowest percentage of lumen in the same season, which was similar to that observed in this species in an Atlantic forest region [28]. This indicates that the highest percentage of seminiferous epithelium observed in the rainy season is associated with a greater proliferation of germline cells in a period of greater food resources [17, 29]. This behavior was repeated in animals inhabiting different biomes.

Considerable differences, however, were observed when comparing morphometric parameters related to the seminiferous tubules in these animals inhabiting the Caatinga, when compared to those of the Atlantic forest. The average tubular diameter presented by the animals in this study was considerably larger when compared both to that found in other bat species and when compared to that found in the same species, in the Atlantic forest. The height of the seminiferous epithelium followed the same trend [15, 16, 17, 24, 28]. In addition, while most mammals usually have an average tubular diameter ranging from 180 to 300 μm [30, 31], the value found here (432.17 μm) is the largest value so far reported. This finding is directly reflected in the length of the seminiferous tubules, which was considerably smaller when compared to the aforementioned animals. While the average tubular length per gram of testis in most mammals is approximately 20 m, and 35 m was previously recorded in *D*. *rotundus*, in this study this value was 6 m [16, 31].

The TSI, on the other hand, was consistent with that found in other animals with polygynic and polyandric mating systems, where in addition to the larger GSI, a large investment is needed specifically in seminiferous tubules, to ensure greater sperm production when compared to animals with a monogamous mating system [17, 24, 25, 32–35].

### Quantification of spermatogenic yield

The cell population present in stage 1 of the SEC in the animals in this study was larger than that found in other bat species, similar to that recorded for *D*. *rotundus* in the Atlantic forest [16, 17, 24], and in general, smaller than those found in other mammals. Each tubular cross-section at stage 1 of SEC in mammals usually exhibit 3.7–8.9 Sertoli cells, 1.1–2.6 A-type spermatogonia, 19.7–21.48 primary spermatocytes in Pre-Leptotene/Leptotene, 17.13–30.60 primary spermatocytes in pachytene, and 54.86–64.01 round spermatids [36–41].

Both the population of Sertoli cells and A-type spermatogonia was higher in the rainy season (3.51±0.74 and 1.00±0.12, respectively), which coincided with the greater development of the seminiferous epithelium in this same season, as previously discussed. These findings were also related to the greater number of Sertoli cells in this season, in order to provide structural and nutritional support to the germline cells [31]. On the other hand, the mitotic index, the general spermatogenic yield and the support capacity performed by Sertoli cells were higher in the dry season (25.43±4.66, 90.79±19.32 and 44.71±22.55, respectively, which may indicate a preparation of seminiferous tubules for an intense sperm production in the subsequent rainy season. In most mammals, including bats, the mitotic index, meiotic index, general spermatogenic yield and Sertoli cells support capacity usually range, respectively, from 11.8–24.8%, 2.5–3.8%, 19.5–74.2 germ cells and 8.5–22 Sertoli cells. The values found in the present study were, in general, higher than the average values found in other mammals, and similar to that found in other bat species [15–17, 24, 31, 39, 41, 42], indicating a high investment on sperm production among the chiropterans.

While the spermatic reserve of the testis and the daily spermatic production of these animals inhabiting the Caatinga, both in the dry and rainy seasons, were considerably lower than those previously seen for *D*. *rotundus* in the Atlantic forest, where the values were, respectively 14.20x10^7^ and 17.25x10^6^ [16], the overall yield of spermatogenesis in the dry season (90.8 cells) was notably higher than previously found for both this species and other bat species (51.4% in *M*. *mololssus*, [25]; 60.0 in *D*. *rotundus*, [16]; 68.7% in *S*. *lilium*, [17]). This indicates that even the number of sperm contained in the testis and the daily spermatic production being lower than reported in the literature, the efficiency of the spermatogenic process as a whole, from A-type spermatogonia to rounded spermatids, was higher in the dry season.

As on the collecting area the dry and rainy seasons stands approximately six months each [12], and the gestational period of *D*. *rotundus* stands seven months [43], the higher mitotic index and overall yield of spermatogenesis in the dry season suggests the occurrence of copulation in this season, in order to allow births to occur at the end of this season or in the beginning of the rainy season, where the puppies will find more favorable conditions for their survival [29, 43]. It also indicates a greater capacity for recovery of the epithelium after the occurrence of spermiation, since well-developed seminiferous epithelium was observed in the rainy season, according to data previously shown.

The total support capacity realized by Sertoli cells, especially in the dry season, of approximately 44 cells, was considerably greater than that observed in other mammals, whose average ranges from 10 to 22 cells [31, 41, 44, 45], indicating a greater efficiency of these cells in *D*. *rotundus* in the Caatinga, since in the Atlantic forest this index was approximately 20 cells [16]. This value is related to the low number of Sertoli cells per gram of testis found in this study (annual average of 6.46 x 10^6^ cells) when compared to other bats (13.10 x 10^7^ in *D*. *rotundus*, [16]; 28.09 x 10^7^ in *M*. *molossus*, [24]; 22.31 x 10^13^ in *S*. *lilium*, [17]), indicating that each Sertoli cell needed to increase its capacity to support germline cells in order to prevent damage to the spermatogenic process in a period of historical water restriction and, consequently, food resources. Thus, although the total support capacity performed by Sertoli cells was greater in the dry season, the smaller individual number of this cell in this same season also indicates the need for these cells to increase their individual support capacity.

### Morphometry and morphology of the intertubular compartment

The elements that compose the intertubular compartment did not vary significantly between the seasons evaluated. Their percentages in the testicular parenchyma, however, were among the lowest values recorded, considering their annual average [18, 31, 40], however close to that previously described for the species in the Atlantic forest [16].

Leydig cells were the main component of the intertubule, as well as found in other bat species [18, 40]. The largest investment in these cells is also directly related to the polygynic mating system presented by *D*. *rotundus*. Therefore, these animals need a greater androgenic investment when compared to monogamous species such as the crab-eating fox and the brown brocket deer [35, 41]. However, the total number of Leydig cells per gram of testis, whose annual average was 47.71 x 10^5^ cells, was far below that found in both *M*. *molossus* (48.49 x 10^6^; [46]) and *S*. *lilium* bats (11.3 x 10^7^ cells; [18]), as well as in *D*. *rotundus* in the Atlantic forest (56.14 x 10^6^; [16]). This parameter was significantly higher in the rainy season, suggesting that in this season high levels of testosterone would be needed to maintain spermatogenesis, which also corroborates previous findings regarding the greater development of the seminiferous epithelium in this same season.

The LSI found in *D*. *rotundus* (0.016%) was similar to that observed in other bats and higher than that observed in larger animals, such as jaguars and ocelots (0.0036%; [47, 48]), which also reinforces the need for greater investment in gamete production in order to maintain harems in these polygynic species [25, 32].

### Ultrastructural and immunohistochemical analyses

The ultrastructural analysis of the cells that make up the stage 1 of the SEC was shown to be similar to that observed in other bat species [49–52]. During spermiogenesis, some unique events occur, including the formation of the acrosome, the condensation of nuclear chromatin, the elimination of residual cytoplasm and the development of the flagellum [53]. Although this process is similar in most animals, in *D*. *rotundus* the pro-acrosome vesicles containing electrodense materials were not observed, which may be related to the absence of a perforatorium, the structure responsible for penetrating the oocyte [54]; therefore, that these electron-dense materials can be the basic substance of their structural organization. In fact, the perforatorium is absent or poorly developed in bats from the families Vespertilionidae [50, 55] and Noctilionidae [56], little developed in Rhinolophidae [57, 58], and well developed in Molossidae [59] and in other Phyllostomidae [49].

The more frequent expression of androgen receptors in the cytoplasm of Leydig cells in the rainy season indicates a synchrony with the peak of spermatogenic activity in that season, as observed in relation to the higher percentage of seminiferous epithelium and number of Leydig cells per gram of testis in this same season, thus proposing a strong link between testosterone and the reproductive cycle in *D*. *rotundus*. This relationship was also observed in the bat *Myotis nigricans*, in which the high expression of androgen receptors was detected in the period of testicular recrudescence, and the period of testicular regression was associated with the low expression of these receptors [60]. It is also known that the expression of androgen receptors in Leydig cells and Sertoli cells is associated with both differentiation of germline cells and spermiogenesis [60–62].

Similarly, aromatase also showed greater expression in the cytoplasm of primary spermatocytes in the preleptotene/leptotene transition in the rainy season, indicating that *D*. *rotundus* obtained the hormonal input of both androgens and estrogens necessary for its spermatogenic process in the period of greater gametes’ production [63].

Fibroblast growth factors (FGFs), especially FGF2, are local regulators that affect meiosis, differentiation and proliferation of germline cells, including the functions of Sertoli cells [64, 65]. Its expression was observed in both spermatocytes and spermatids of *D*. *rotundus* in the two seasons evaluated, as well as in other animals, which corroborates its role in maintaining the integrity of the seminiferous epithelium [66].

The anti-apoptotic protein BCL-2 is one of the most important regulators of the intrinsic pathway of apoptosis. Its expression in the testicles of *D*. *rotundus* was similar to that observed in the rodent *Lagostomus maximus*, in which the expression of BCL-2 was detected only in spermatocytes and rounded spermatids, during the active period of spermatogenesis [67]. The similar expression of both FGF2 and BCL-2 throughout the dry and rainy seasons indicates the absence of a testicular regression period in *D*. *rotundus* in the Caatinga Biome.

## Conclusions

It can be concluded that *D*. *rotundus* showed greater development of the seminiferous epithelium during the rainy season and a higher overall yield of spermatogenesis during the dry season. The morphometric findings of the animals sampled in this study, which were inhabitants of the Caatinga Biome, showed marked differences in relation to individuals of the same species, which were inhabitants of the Atlantic Forest Biome. However, the morphometry of the germline cells, Sertoli cells and Leydig cells, associated with the relatively constant expression of androgen receptors, aromatase, FGF2 and BCL-2 throughout the year, allowed the continuation of spermatogenesis without a period of reproductive inactivity, even in periods of scarce food resources, as in the dry season, although sperm production is higher in the rainy season.
